# Bone Marrow Stem Cells Derived from Nerves Have Neurogenic Properties and Potential Utility for Regenerative Therapy

**DOI:** 10.3390/ijms24065211

**Published:** 2023-03-08

**Authors:** Leah C. Ott, Christopher Y. Han, Jessica L. Mueller, Ahmed A. Rahman, Ryo Hotta, Allan M. Goldstein, Rhian Stavely

**Affiliations:** Department of Pediatric Surgery, Massachusetts General Hospital, Harvard Medical School, Boston, MA 02114, USA

**Keywords:** bone marrow, Schwann cell, stem cell, neural stem cell, neural crest, neural crest stem cell, neurovascular, enteric nervous system

## Abstract

Neurons and glia of the peripheral nervous system are derived from progenitor cell populations, originating from embryonic neural crest. The neural crest and vasculature are intimately associated during embryonic development and in the mature central nervous system, in which they form a neurovascular unit comprised of neurons, glia, pericytes, and vascular endothelial cells that play important roles in health and disease. Our group and others have previously reported that postnatal populations of stem cells originating from glia or Schwann cells possess neural stem cell qualities, including rapid proliferation and differentiation into mature glia and neurons. Bone marrow receives sensory and sympathetic innervation from the peripheral nervous system and is known to contain myelinating and unmyelinating Schwann cells. Herein, we describe a population of neural crest-derived Schwann cells residing in a neurovascular niche of bone marrow in association with nerve fibers. These Schwann cells can be isolated and expanded. They demonstrate plasticity in vitro, generating neural stem cells that exhibit neurogenic potential and form neural networks within the enteric nervous system in vivo following transplantation to the intestine. These cells represent a novel source of autologous neural stem cells for the treatment of neurointestinal disorders.

## 1. Introduction

The neural crest (NC) is an important embryonic compartment that gives rise to neurons and glia of the peripheral nervous system (PNS). Notable lineages of NC-derived cells in the embryonic environment include enteric NC-derived cells that form the enteric nervous system (ENS), including enteric glia and neurons, and the embryonic Schwann cell precursors that differentiate into myelinating and unmyelinating Schwann cells, somatic sensory neurons, autonomic neurons, and melanocytes [1,2,3,4,5]. NC-derived cells of the PNS that are isolated from the postnatal microenvironment have also been shown to exhibit neural stem cell (NSC) qualities, including rapid proliferation and differentiation into mature glial cells and neurons [5,6]. These NSCs have been found to originate from the nervous system niche and arise from glial cells expressing Plp1, including the enteric glia of the intestine and Schwann cells of the subcutaneous adipose tissue (SAT) [7,8]. The unique properties of these progenitor populations provide an intriguing prospect for regenerative therapies for disorders affecting the central nervous system (CNS), PNS, and ENS.

Bone marrow (BM) is innervated by the PNS, with primarily sensory and sympathetic nerve fibers running within the medullary cavity [9,10]. Interest in this neural signaling network has increased over the last several years, with recent studies elucidating that these neurons regulate hematopoietic stem cell (HSC) mobilization and quiescence [10,11,12]. The nerve fibers of BM are supported by closely apposed Schwann cells, which can be identified around myelinated and unmyelinated axons [9,10,12]. Unmyelinating Schwann cells appear to reside adjacent to the vasculature of BM [12,13]. Overall, our understanding of Schwann cell populations in BM is limited, but recent studies suggest their functions extend beyond support of the nervous system, with a paracrine role in the maintenance of the HSC niche [10,12]. 

BM is widely regarded as a source of two progenitor cell populations as follows: HSCs, which give rise to all circulating myeloid and lymphoid cells; and mesenchymal stem cells (MSCs). Both populations are easily isolated and expanded from BM aspirates and have been administered to patients in the clinical setting [14,15]. Interestingly, MSCs have been reported to spontaneously transdifferentiate into cells with Schwann cell characteristics [16]. Nevertheless, our previous work on the SAT raises the prospect that observations of neuronal differentiation in MSCs is a potential result of a contaminating pool of Schwann cell-derived NSCs that become highly plastic during in vitro culture conditions [7]. Further evidence of the stem cell properties of Schwann cells can also be observed in vivo with reports of Schwann cells giving rise to enteric neurons in the postnatal gut environment [5]. Therefore, we hypothesize that a population of Schwann cells with NSC properties may exist in BM, similar to those we previously described in the intestine and SAT [7,8]. 

In this study, we characterize Schwann cells residing in BM. Using this knowledge, Schwann cells are isolated and cultured to assess their NSC-like qualities with in vitro assays and in vivo transplantation studies of the intestine, demonstrating their potential for clinical application as regenerative therapies, particularly for neurointestinal diseases. 

## 2. Results

### 2.1. Schwann Cells Reside along Nerve Fiber Projections in the Bone Marrow in Close Apposition to Blood Vessels

**Figure 1:**
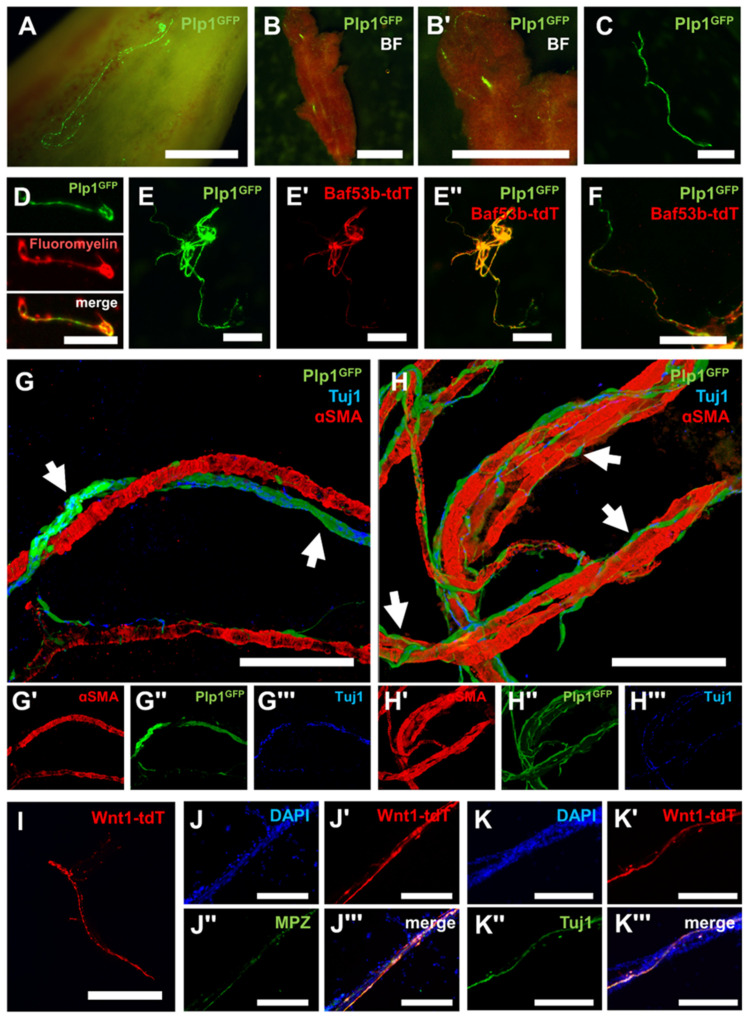
Neural crest-derived Schwann cells reside in the perivascular space of bone marrow in association with nerve fibers. (**A**) Representative high magnification image of the endosteum of the femur of Plp1^GFP+^ mice. Scale bar = 1 mm. Representative low (**B**) and high (**B′**) magnification images of bone marrow (BM) from Plp1^GFP+^ mice. Scale bars = 2 mm. (**C**) Free-floating, fiber-like structure after trituration of BM from Plp1^GFP+^ mice. Scale bar = 1 mm. (**D**) FluoroMyelin Red staining of a myelinated Plp1^GFP+^ nerve fiber from triturated BM. Scale bar = 200 µm. (**E**–**E′′**) Representative images of nerve fiber bundles (NFBs) from triturated BM expressing Plp1^GFP^ (**E**), Baf53b-tdT (**E′**), and merged image (**E′′**). Scale bar = 1 mm. (**F**) High magnification image of the previous demonstrating individual nerve fiber projections. Scale bar 500 µm. (**G**–**G′′′**) Representative images of immunocytochemistry of BM from Plp1^GFP+^ mice with Schwann cells in NFBs (arrows) colocalizing with nerve fibers (Tuj1) in close proximity to arterioles (αSMA) (**G**), bottom panels show the expression of αSMA (**G′**), Plp1^GFP^ (**G′′**), and Tuj1 (**G′′′**) independently. (**H**–**H′′′**) Representative images demonstrating individual Schwann cells intermittently lining nerve fiber projections (arrows) and wrapping tightly around blood vessels, bottom panels show the expression of αSMA (**H′**), Plp1^GFP^ (**H′′**), and Tuj1 (**H′′′**) independently. (**G**,**H**) Images are 3D-rendered using ImageJ. Scale bars = 100 µm. (**I**) Free-floating, fiber-like structure after trituration of BM from Wnt1-tdT^+^ mice. Scale bar = 1 mm. (**J**–**J′′′**) Visualization of DAPI-stained nuclei (**J**), Wnt1-tdT, (**J′**), Myelin protein zero (MPZ) (**J′′**), and merged image (**J′′′**) in NFBs isolated from the BM. (**K**–**K′′′**) Visualization of DAPI-stained nuclei (**K**), Wnt1-tdT (**K′**), Tuj1 (**K′′**), and merged image (**K′′′**) in NFBs isolated from the BM. (**J**,**K**) Scale bars = 200 µm.

To characterize the presence and localization of Schwann cells within BM, Plp1^GFP+^ transgenic mice with pan-glial promoter-driven expression of GFP were utilized (Figure 1A) [17]. Femurs were collected, bisected in the sagittal plane, and BM was flushed to expose the endosteum, with fiber-like structures of Plp1^GFP+^ cells observed running parallel to the long axis of the bone (Figure 1A). Fiber-like structures containing Plp1^GFP+^ cells were also observed within the intact BM plug after flushing the medullary cavity of the femur (Figure 1B). Gentle trituration of intact BM was sufficient to liberate the Plp1^GFP+^ fiber-like structures into a free-floating suspension (Figure 1C). Positive staining of these Plp1^GFP+^ structures with FluoroMyelin Red indicated that these structures are Schwann-cell-containing nerve fibers (Figure 1D). To further validate the notion that these structures represent nerve fibers, Plp1^GFP+^; Baf53b-tdT^+^ dual neuro-glial reporter mice were utilized [7,17,18]. Plp1^GFP+^ cells were confirmed to be expressed in the same structures as neuronal Baf53b-tdT^+^ nerve fibers (Figure 1E). Higher magnification images demonstrated that Plp1^GFP+^ cells were positioned along individual Baf53b-tdT^+^ nerve fiber projections, confirming their Schwann cell identity (Figure 1F). These results were validated in samples from Plp1^GFP+^ mice with nerve fibers visualized by the nerve fiber marker Tuj1 (TUBB3). Nerve fiber bundles (NFBs) of Plp1^GFP+^ Schwann cells and Tuj1^+^ nerve fibers (arrows) were found to travel in close proximity and were largely parallel to arterioles in BM as identified by alpha-smooth muscle actin (α-SMA) (Figure 1G). High magnification images also indicated smaller individual branches of Tuj1^+^ nerve fibers lined with intermittent Plp1^GFP+^ Schwann cells (arrows) wrapping tortuously around blood vessels in tight apposition (Figure 1H). Transgenic Wnt1-tdT^+^ NC-derived cell tracer mice were then used to confirm the embryonic origin of these structures [7,19,20]. Fiber-like structures similar to the Plp1^GFP+^ mice were observed after gentle trituration of BM (Figure 1I). These Wnt1-tdT^+^ structures expressed the myelinating Schwann cell marker myelin protein zero (MPZ) and Tuj1, confirming that Schwann cells and nerve fibers, respectively, in BM are NC-derived (Figure 1J,K).

### 2.2. Schwann Cells Acquire Neural Stem Cell Properties In Vitro

**Figure 2:**
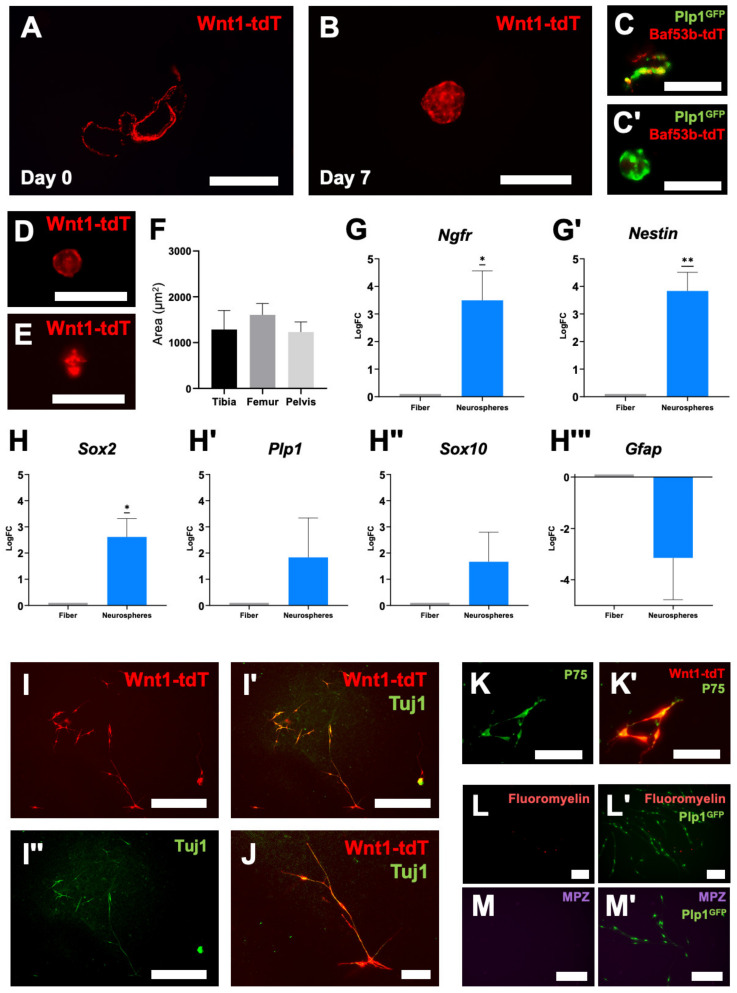
Neural crest-derived Schwann cells from bone marrow develop neural stem cell features in vitro. Expression of Wnt1-tdT in free-floating nerve fibers (**A**) from BM (day 0) and BM-neural stem cell (BM-NSC) neurospheres (**B**) in culture (day 7). Scale bars = 1 mm. Expression of Plp1^GFP^ and Baf53b-tdT in free-floating nerve fibers (**C**) and BM-NSC neurospheres (**C′**) after 7 days in culture. Scale bars = 100 µm. Representative images of BM-NSC neurospheres generated from the pelvis (**D**) and tibia (**E**) of Wnt1-tdT^+^ mice. Scale bars = 200 µm. (**F**) Quantification of BM-NSC neurosphere size by source. One-way ANOVA with Welch’s correction (not statistically significant). Tibia, *n* = 7 independent cultures; Femur, *n* = 6 independent cultures; Pelvis, *n* = 3 independent cultures. (**G**,**G′**) Quantitative PCR of *Ngfr* (**G**) and *nestin* (**G′**) gene expression in NFBs and neurospheres. One-sample *t*-test to LogFC of 0, *n* = 7, * *p* < 0.05, ** *p* < 0.01. (**H**–**H′′′**) Quantitative PCR of *Sox2* (**H**)*, Plp1* (**H′**), *Sox10* (**H′′**) and *Gfap* (**H′′′**) gene expression in NFBs and neurospheres. One-sample *t*-test to LogFC of 0, * *p* < 0.05. *Sox2* and *Plp1, n* = 5; *Sox10*, *n* = 7; *Gfap*, *n* = 6. (**I**–**I′′**) Representative images of Wnt1-tdT^+^ BM-NSCs cultured on fibronectin in monolayer conditions (**I**), merged images with Tuj1 expression (**I′**), and Tuj1 expression shown independently (**I′′**). Scale bar = 500 µm. (**J**) High magnification image of the above. Scale bar = 100 µm. (**K**,**K′**) Expression of P75 (**K**) in Wnt1-tdT^+^ BM-NSCs (**K′**). Scale bar = 50 µm. (**L**–**M′**) Representative images of FluoroMyelin (**L**) and MPZ expression (**M**) in Plp1^GFP^ expressing BM-NSCs (**L′**,**M′**). Scale bars = 200 µm.

Using the methods described above, nerve fiber structures were isolated from Wnt1-tdT^+^ mice and cultured in vitro in neuroproliferation media (Figure 2A). After 7 days, these structures generated free-floating neurospheres containing Wnt1-tdT^+^ cells (Figure 2B). Repetition of these experiments in the Plp1^GFP+^; Baf53b-tdT^+^ dual neuro-glial reporter mice indicated that Baf53b-tdT^+^ structures lose tdT expression during the culture period, leaving Plp1^+^ cells to form neurospheres (Figure 2C). This is consistent with an extrinsic origin of Baf53b-tdT^+^ nerve fibers, which are no longer supported once severed from their neuronal cell bodies located externally to BM. Wnt1-tdT^+^ and Plp1^GFP+^ fiber-like structures were obtained from several different sources of bone after flushing and trituration, including the pelvis (Figure 2D), femur, and tibia (Figure 2E). All sources had similar capabilities for neurosphere formation (Figure 2F), although the lower extremity long bones were the most amenable to isolation due to their relatively large size and long medullary cavity that is easily accessed with a straight needle.

The expression of stem cell and glial markers was compared between the initial fiber structures and the resulting neurospheres after 7 days in culture using quantitative PCR (qPCR) (Figure 2G,H). The NC stem cell marker *Ngfr* and NSC marker *nestin* were upregulated in the neurospheres compared to fibers, suggesting that these cells acquire NSC qualities in culture (Figure 2G). *Sox2*, which is highly expressed in embryonic NSCs, was also upregulated (Figure 2H). No statistical differences were observed in glial/Schwann cell markers between the fibers and neurospheres, including *Plp1*, *Sox10*, and *Gfap* (Figure 2H). There was, however, a trend in increasing expression of *Plp1* and *Sox10*, which could be consistent with the relative expansion of the glia/Schwann-cell-derived population in the neurospheres. Neurospheres were then cultured on fibronectin in monolayer conditions for 7 days. Wnt1-tdT^+^ cells, which are NC-derived, were the only cells to express the neuronal marker Tuj1 in these conditions (Figure 2I,J). Furthermore, these Wnt1-tdT^+^ cells were confirmed to express NC stem cell marker P75 (NGFR) (Figure 2K). Together, these data indicate that NSCs derived from BM (BM-NSCs) can be isolated from nerve fibers and acquire neurogenic potential in vitro. Unlike our observations in the initial NFBs, the Plp1^+^ cells from neurospheres no longer colocalized with FluoroMyelin Red or expressed MPZ, suggesting the myelinating Schwann cell phenotype is not retained by this BM-NSCs population (Figure 2L,M).

### 2.3. Bone Marrow Neural Stem Cells Integrate with the Enteric Nervous System in the Gut

**Figure 3:**
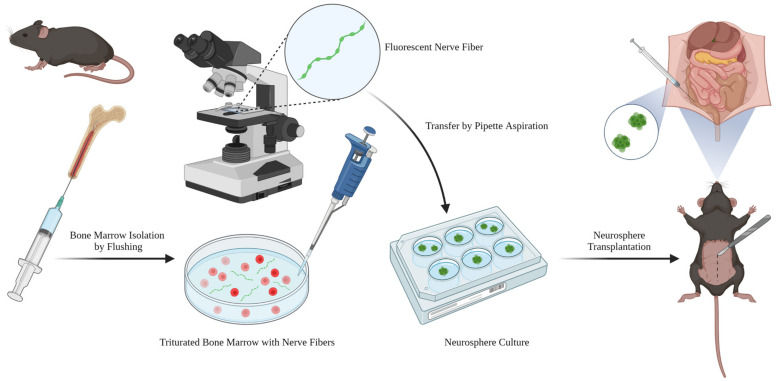
Overview of BM nerve fiber isolation, culturing, and neurosphere transplantation. Adult mice were sacrificed, and the femur, tibia, and pelvis dissected. The BM plug was liberated by flushing the medullary cavity with PBS, then gently triturated to free the nerve fibers from the stroma. Nerve fibers were identified by fluorescent microscopy and transferred to neuroproliferation media for culture by pipette aspiration. For in vivo transplantation experiments, neurospheres were surgically implanted into the colon or colorectum via injection with a microliter syringe.

**Figure 4:**
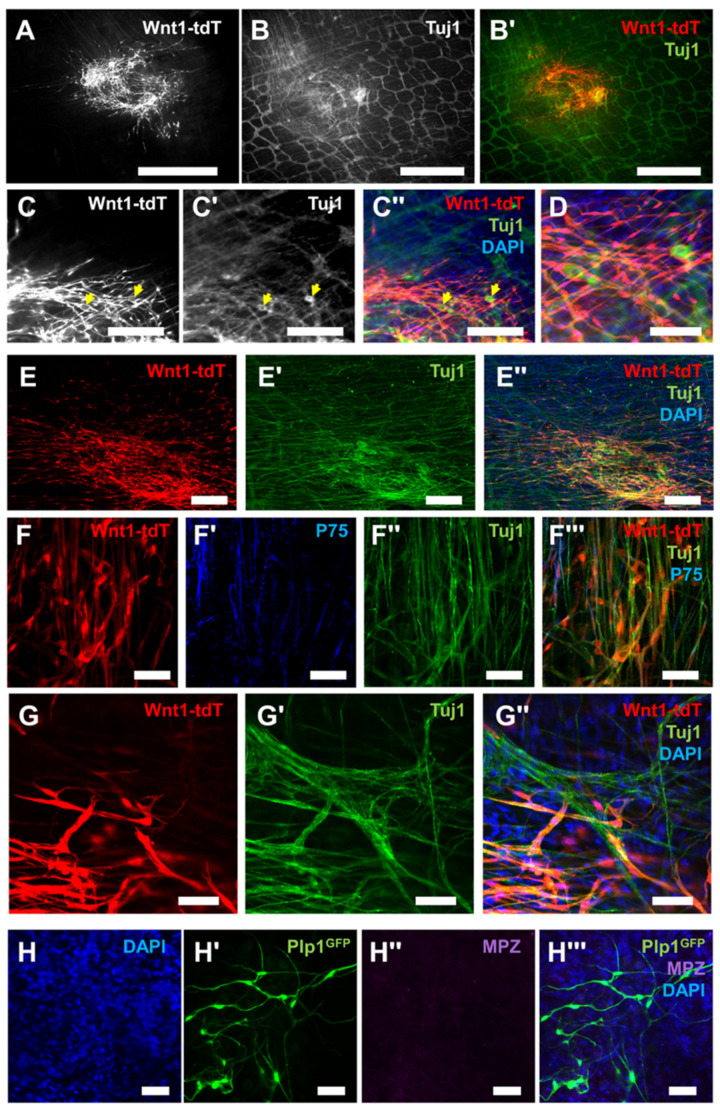
Bone marrow neural stem cells migrate, differentiate into neurons, and engraft with the enteric nervous system after in vivo transplantation. (**A**) Representative image of Wnt1-tdT^+^ BM-NSC migration and cell spread in the mid-colon at 8 weeks post-implantation. Scale bar = 1 mm. (**B**,**B′**) Images of whole-mount preparations of recipient longitudinal muscle myenteric plexus (LMMP) with Tuj1 (**B**) expression in transplanted Wnt1-tdT^+^ NSCs (**B′**). Scale bar = 1 mm. (**C**–**C′′**) Representative images of transplanted Wnt1-tdT^+^ NSCs (**C**), Tuj1 expression (**C′**), and merged image (**C′′**) in whole-mount preparations. Yellow arrows indicate Tuj1^+^ neuronal soma derived from BM-NSCs. Scale bar = 250 µm. (**D**) High magnification image of the above. Scale bar = 100. (**E**–**E′′**) Representative images of the colorectum whole-mount preparations at 3 weeks post-transplantation containing Wnt1-tdT^+^ BM-NSCs (**E**), Tuj1 expression (**E′**), and merged image containing DAPI-stained nuclei (**E”**). Scale bar = 200 µm. (**F**–**F′′**) Images of Wnt1-tdT^+^ BM-NSCs (**F**) forming interconnected clusters of cells similar to ganglia, expressing P75 (**F′**), Tuj1 (**F′′**), and merged image (**F′′′**). Scale bar = 50 µm. (**G**–**G″**) Images at the periphery of the implantation site showing Wnt1-tdT^+^ BM-NSCs (**G**), expression of Tuj1 (**G′**), and merged image including DAPI-stained nuclei (**G″**). Wnt1-tdT^+^ BM-NSCs co-expressed Tuj1 and form connections with, and incorporate into, the host ENS. Scale bar = 50 µm. (**H**–**H′′′**) Representative images of DAPI-stained nuclei (**H**), transplanted Plp1^GFP+^ BM-NSCs (**H′**), MPZ expression (**H′′**), and merged image (**H′′′**) depicting that BM-NSCs do not regain the expression of the myelinating Schwann cell protein MPZ after transplantation to the gut. Scale bar = 50 µm.

To examine the potential of these neurogenic BM cells for regenerative applications, transplantation experiments were conducted on the muscularis externa of the colon in mice, which contains the myenteric plexus of the ENS, as summarized in Figure 3. Neurospheres generated from Wnt1-tdT^+^ mice were surgically implanted to the muscularis externa of the mid-colon by laparotomy in mice under general anesthesia. After 8 weeks, Wnt1-tdT^+^ cells migrated and spread within the muscularis externa and expressed the neuronal marker Tuj1, as did the enteric neurons of the recipient (Figure 4A,B). Neuronal soma comparable in morphological appearance to enteric neurons could be identified, originating from the transplanted Wnt1-tdT^+^ cells (Figure 4C,D). Further experiments were conducted by implanting neurospheres into the muscularis externa of the colorectum via a surgical transanal approach. Mice sacrificed at 3 weeks after transplantation similarly demonstrated successful engraftment of Wnt1-tdT^+^ cells, which extended projections and expressed Tuj1 (Figure 4E). High magnification images indicated that Wnt1-tdT^+^ cells express P75 or Tuj1 and aggregate into small clusters that interconnect with one another in formations reminiscent of enteric ganglia (Figure 4F). At the periphery of the transplantation site, Wnt1-tdT^+^ cells formed physical connections and were incorporated into the host’s endogenous enteric ganglia, suggesting successful integration with the recipient ENS (Figure 4G). Transplanted cells did not appear to reacquire the myelinating characteristics of their Schwann cell progeny, as indicated by a lack of MPZ staining in transplanted cells from Plp1^GFP+^ mice (Figure 4H).

## 3. Discussion

The present study describes a population of Schwann cells residing along nerve fibers in BM in close proximity to the vasculature. These Schwann cells can be easily isolated and cultured, demonstrating plasticity in vitro and forming neurospheres that express NSC markers. With continued culture on fibronectin or transplantation to the intestine in vivo, they begin to express neuronal markers, but not myelinating Schwann cell markers. Following transplantation of these cells to the intestine in vivo, they migrate, differentiate into neurons, and integrate with the host ENS, suggesting potential for regenerative therapy application as a novel source of autologous NSCs.

BM is innervated by sensory and autonomic neurons and contains both myelinating and unmyelinating Schwann cells [9,10,12,13]. Consistent with our observations, Thai et al. described a perivascular network of unmyelinating, NC-derived Schwann cells closely associated with sensory and sympathetic nerve fibers in the BM of neonatal and adult mice [13]. Autonomic signaling plays a critical regulatory role in HSC trafficking during homeostasis and acute stress, with norepinephrine from sympathetic neurons stimulating the coordinated release of HSCs into the periphery in accordance with the circadian rhythm [10,11]. Nestin^+^ mesenchymal cells were originally thought to mediate the effect of adrenergic signaling on HSCs, but Yamazaki et al. recently described a population of unmyelinating Schwann cells in BM associated with autonomic nerve fibers that were responsible for maintaining HSC dormancy via the TGFβ/SMAD signaling pathway [10,12,21]. Furthermore, a population of Nestin^+^ BM cells, namely Schwann cell precursors and MSCs, were shown to recruit HSCs to long bones during embryonic development [22]. These studies support a critical role of BM Schwann cells in establishing and maintaining the HSC niche, but the neurogenic properties of these cells have not been previously reported [12,22].

Growing evidence from the fields of neurodevelopment and neurodegenerative disease supports a critical role for the neurovascular niche, composed of neurons, glia, axons, and endothelial cells of blood vessels, in homeostatic and disease states [23,24,25,26,27,28,29]. During the development of the PNS, blood vessels frequently migrate alongside peripheral nerves, directed by shared guidance signals, and each release mediators that support the survival and branching of the other [30,31,32,33,34,35]. Time-lapse studies of embryonic development of the PNS utilizing in vivo imaging subsequently found that migrating NC cells are closely apposed to endothelial cells, with the latter modifying the trajectory of the former through direct cell–cell contact during the creation of sympathetic and dorsal root ganglia (DRG) [36]. The neurovascular unit of the CNS is composed of neurons, glia, pericytes, and vascular endothelial cells, which participate in crosstalk critical to the maintenance and survival of neurons and endothelial cells, as well as establish a niche for NSCs residing near the vasculature [24,37,38]. Brain pericytes are derived from both the mesoderm and NC and have demonstrated neurogenic and gliogenic properties comparable to NC stem cells [39,40,41,42,43,44,45]. NC-derived pericytes have been identified in the perivascular niche of other tissues, including the spinal cord, thymus, muscle, and dermis, but seem to favor differentiation into mesenchymal lineages (such as osteoblasts, adipocytes, and chondroblasts) over neural lineages [46,47,48,49,50]. Interestingly, Xu et al. described a population of NGFR^+^ cells residing in the perivascular space of the tendon that expresses various NC-derived stem cell markers (including Vimentin, Snail, and Sox10) and demonstrated neurogenic potential in vitro [51].

We found that perivascular, NC-derived Schwann cells isolated from BM acquired NSC properties in vitro, similar to SAT-derived Schwann cells and enteric-derived glia previously described by our group [7,8]. It is unclear whether this plasticity represents a capacity for neural differentiation ubiquitous to Schwann cells in all postnatal NFBs or if artificial conditions in vitro promote dedifferentiation. Expression of NC-derived stem cell and NSC markers, *Ngfr* and *nestin*, were markedly increased in BM-NSCs following cell culture. This could represent in vitro dedifferentiation processes analogous with the formation of SAT-NSCs from Schwann cells [7]. Further study of such pathways in BM-derived Schwann cells and NSCs is critical to fully characterize the mechanism of NSC formation.

Previously, Nagoshi et al. reported that NC-derived stem cells could be isolated from BM using Wnt1-cre reporter mice [44]. These cells are capable of generating spheroids and differentiating into neurons, glia, and myofibroblasts in vitro, demonstrating the high plasticity of this cell population [44]. Our data suggest that this NC stem cell population likely arises from Plp1^+^ Schwann cells, which would explain their gliogenic and neurogenic affinity. It is unclear whether a similar population of cells with neurogenic potential exists in human BM. However, Coste et al. described a population of stem cells isolated from healthy human subjects that could generate spheroids expressing Nestin and NGFR with adipogenic, osteogenic, chondrogenic, gliogenic, and neurogenic differentiation potential in vitro [52]. The authors suggested that these cells would not be distinguished by the cell-surface expression panel commonly used to identify MSCs [52]. Furthermore, Brboric et al. identified and purified a subset of NGFR^+^ stem cells from BM aspirates of healthy adults, which formed neurospheres in vitro and could migrate and differentiate into neurons, glia, and myofibroblasts [53]. Notably, BM-MSCs have been reported to transdifferentiate into cells with Schwann cell characteristics in a spontaneous manner [16]. It is possible that MSC cultures could be contaminated by NC-derived Schwann cells with gliogenic and neurogenic plasticity in vitro.

Our BM-NSCs acquired neuronal features in vitro and in vivo following transplantation into the colon, resulting in Tuj1 expression and an associated loss of FluoroMyelin Red and MPZ positivity. Based on these observations, we hypothesize that the fate of BM-NSCs is influenced by their environment. Schwann cells from mice and zebrafish are known to differentiate into enteric glia and neurons upon arrival to the intestine postnatally and culturing human NC stem cells derived from induced pluripotent stem cells (iPSCs) with gut tissue explants promotes their differentiation to enteric neurons [5,6,54]. SAT-NSCs derived from Schwann cells also exhibit neuronal differentiation following ex vivo and in vivo transplantation into the intestine, further supporting the role of the microenvironment in the fate commitment of different NSC populations [7]. Finally, Shea et al. described a population of Nestin^+^ cells in BM that differentiated into Schwann cells in vitro in the presence of DRG neurons in a direct cell–cell contact-dependent manner [55]. They termed these BM cells in culture “Schwann cell-like cells”. This may be the same population of Schwann cell-derived BM-NSCs described in our study.

Autologous ENSCs and SAT-NSCs have been proposed as potential stem cell therapies for the treatment of various disorders, including injuries of the CNS and PNS and congenital and acquired disorders of the ENS [7,19,56]. Transplanted NSCs have been shown to survive, migrate, and develop into functional neuronal networks in murine models of gastroparesis and Hirschsprung disease (HSCR) by our group and others [7,19,57,58]. BM-NSCs could represent another source of autologous NSCs and may be particularly suited for the treatment of neurointestinal disorders, including esophageal achalasia, gastroparesis, and HSCR, given their propensity for neurogenesis in the intestinal microenvironment. An attractive feature of these BM-NSCs is ease of access through BM aspiration or biopsies, enabling the generation of autologous cells following a minimally invasive procedure. Whether they can be expanded in sufficient numbers in vitro, however, requires further investigation.

One limitation of this study is that we did not demonstrate that BM-NSCs generate functional neuronal networks following in vivo transplantation, as was previously demonstrated with SAT-NSCs and ENSCs in murine models of HSCR and gastroparesis [7,19,57,58]. Additionally, we do not know whether similar NC-derived Schwann cells are present in human BM samples nor whether they can be easily purified, cultured, and implanted in vivo. Human BM samples should be evaluated for the presence of homologous Schwann cells, and their isolation, culturing, and transplantation attempted to demonstrate feasibility for future clinical applications. Additionally, future studies should investigate transplantation of BM-NSCs into models of gastrointestinal dysmotility to determine whether they generate functional neuronal networks and restore gut motility.

In summary, we describe a population of NC-derived Schwann cells residing in a neurovascular niche in BM. These Schwann cells demonstrate plasticity and generate BM-NSCs that exhibit neurogenic potential in vitro and in vivo. Given the accessibility of BM in the clinical setting, these cells may represent a novel source of autologous NSCs for the treatment of neurointestinal disorders.

## 4. Methods

### 4.1. Study Design

This study was designed to determine whether NC-derived Schwann cells are present in the BM of mice, as has been previously described in the SAT, and whether they can generate NSCs [7]. All study protocols were IACUC (2009N000239) approved.

### 4.2. Mice

Wnt1::Cre (Tg(Wnt1-Cre)11Rth, JAX Stock #003829) [7,19,20], BAF53b::Cre (Tg(Act16b-Cre)4092Jiwu/J, JAX Stock #027826) [7,18], and ROSA26^tdTomato^ (ROSA26R-tdTomato reporter, JAX Stock #007914) [7,19,20] mice were purchased from the Jackson Laboratory. Plp1^GFP+^ mice were kindly gifted by W. Macklin, University of Colorado [17]. All mice were housed and bred at the Center for Comparative Medicine animal facility at Massachusetts General Hospital under specific pathogen-free conditions. All experiments were approved by the Institutional Animal Care and Use Committee of Massachusetts General Hospital.

### 4.3. Nerve Fiber Isolation Procedure

Adult mice (6 to 10 weeks old) were sacrificed by CO_2_ asphyxiation. The tibia, femur, and pelvis were dissected from these mice, stripped of all attached muscles and tendons, and washed in sterile phosphate-buffered saline (PBS). The epiphyses of the long bones and the inferior pubic rami of the pelvis were sharply transected to expose the medullary cavity. The medullary cavity was flushed with sterile PBS using a 25-gauge needle, liberating BM. BM was triturated in sterile PBS to dissociate the nerve fibers from surrounding stromal cells. These tdT^+^ or GFP^+^ fibers were visualized using a Leica MZ FLIII fluorescence stereomicroscope (Leica Microsystems, Inc., Deerfield, IL, USA), aspirated with a sterile pipette, and transferred to 4% paraformaldehyde for immunocytochemistry or neuroproliferation media for neurosphere culture.

### 4.4. Neurosphere Culture

Nerve fibers isolated as above were seeded in 24-well low-attachment tissue culture plates in 0.5 mL of neuroproliferation media. Neuroproliferation media contained penicillin and streptomycin (1%; Life Technologies, Thermo Fisher Scientific, Waltham, MA, USA), B27 supplement (1×; Gibco, Thermo Fisher Scientific), N-2 Supplement (1x; Gibco, Thermo Fisher Scientific), basic fibroblast growth factor (20 ng/mL; Stemcell Technologies, Vancouver, BC, Canada), insulin-like growth factor 1 (20 ng/mL; Thermo Fisher Scientific), retinoic acid (75 ng/mL; Sigma Aldrich, St. Louis, MO, USA), and 2-mercaptoethanol (50 μmol/L; Gibco, Thermo Fisher Scientific) in equal parts Neurocult Mouse Basal Medium (Stemcell Technologies) and Dulbecco’s Modified Eagle Medium (DMEM; Gibco, Thermo Fisher Scientific). Cells were cultured under sterile conditions in a humidified incubator at 37 °C with 5% CO_2_ and atmospheric oxygen. Cells remained in culture for 7 days, at which time spheroids were present.

Spheroid-containing media were transferred to 12-well tissue culture plates coated in fibronectin (1:100 in sterile PBS for 2 h at 37 °C), with an additional 0.5 mL of neuroproliferation media for a final volume of 1 mL per well to promote spheroid attachment. After 7 days in culture as described above, the media were discarded, and cells were fixed in 4% paraformaldehyde for immunocytochemistry.

### 4.5. Quantitative PCR

Nerve-fiber- or spheroid-containing media were transferred to a microcentrifuge tube and centrifuged at 500× *g* for 5 min. The supernatant was discarded, and the nerve fibers or neurospheres were resuspended in an RLT buffer (Qiagen, Germantown, MD, USA) and stored at −80 °C until time of analysis. Total RNA was extracted from these nerve fibers and neurospheres with an RNeasy Mini kit (Qiagen). The concentration of extracted RNA was quantified using the Qubit RNA HS Assay Kit (Invitrogen, Thermo Fisher Scientific) on a Qubit 4 Fluorometer (Invitrogen, Thermo Fisher Scientific, Waltham, MA, USA). Total RNA was reverse-transcribed and amplified via RT-qPCR with the iTaq Universal SYBR Green One-Step Kit (Bio-Rad, Hercules, CA, USA) using a Bio-Rad CFX96 real-time thermal cycler. The reaction setup was performed as per the manufacturer’s instructions. *Gapdh* was amplified using the primer sequences (5′→3′) AGGTCGGTGTGAACGGATTTG (forward) and TGTAGACCATGTAGTTGAGGTCA (reverse). *Gfap* was amplified using the primer sequences (5′→3′) GGGGCAAAAGCACCAAAGAAG (forward) and GGGACAACTTGTATTGTGAGCC (reverse). *Nestin* was amplified using the primer sequences (5′→3′) CCCACCTATGTCTGAGGCTC (forward) and GGGCTAAGGAGGTTGGATCAT (reverse). *Ngfr* (*P75*) was amplified using the primer sequences (5′→3′) CCTGGACAGTGTTACGTTCTC (forward) and ACACAGGGAGCGGACATACT (reverse). *Plp1* was amplified using the primer sequences (5′→3′) TGAGCGCAACGGTAACAGG (forward) and GGGAGAACACCATACATTCTGG (reverse). *Sox2* was amplified using the primer sequences (5′→3′) GCGGAGTGGAAACTTTTGTCC (forward) and CGGGAAGCGTGTACTTATCCTT (reverse). *Sox10* was amplified using the primer sequences (5′→3′) CGGACGATGACAAGTTCCCC (forward) and GTGAGGGTACTGGTCGGCT (reverse). The thermal cycling protocol was carried out as per the kit manufacturer’s instructions. Results were processed using Bio-Rad CFX Manager software (version 3.1), with a standard threshold to quantity cross point (Ct) values. Ct values for *Ngfr*, *nestin*, *Sox2*, *Sox10*, *Plp1*, and *Gfap* were normalized to *Gapdh* expression in each sample as an internal control. All reactions were performed in triplicate.

### 4.6. In Vivo Neurosphere Transplantation

To examine whether allogenic BM-NSCs can survive, differentiate, and incorporate with the host ENS in the gastrointestinal tract, neurospheres were transplanted into 8-week-old wild-type mice. Neurospheres were generated by seeding Wnt1-tdT^+^ or Plp1^GFP+^ nerve fibers (isolated as above) in 24-well low-attachment tissue culture plates in 0.5 mL neuroproliferation media. Neurospheres were then collected for transplantation after 12 days in culture. Neurospheres were surgically implanted in either the mid-colon via laparotomy or colorectum via a transanal approach, as previously reported [7,58,59]. Briefly, general anesthesia was induced with inhaled isoflurane (1–4%; Covetrus, Portland, ME, USA). For mid-colon transplantation, a laparotomy was performed to expose the colon. Neurospheres were implanted between the longitudinal and circular muscle layers of the colon by injection through its serosal surface with a microliter syringe with a 33- or 36-gauge needle. For colorectum transplantation, two 5-0 silk stay sutures were placed in the rectal mucosa. These sutures were then gently retracted towards the anal verge, prolapsing the colorectum. Neurospheres were implanted between the longitudinal and circular muscle layers of the colorectum by injection through its mucosal surface with a microliter syringe with a 33- or 36-gauge needle. For all surgeries, one to two neurospheres were implanted in the colon, as previously reported [7,58].

### 4.7. Immunocytochemistry for In Vitro Experiments

Adult mice (6 to 10-weeks-old) were sacrificed by CO_2_ asphyxiation. Neuronal fibers and adherent cells in monolayer culture were isolated as described above and fixed in 4% paraformaldehyde at room temperature for at least 30 min.

FluoroMyelin staining of nerve fibers and adherent cells was performed using FluoroMyelin Red Fluorescent Myelin Stain (1:300, Invitrogen) in Neurocult Mouse Basal Medium (Stemcell Technologies) for 20 min, followed by three washes in PBS. For all other immunocytochemistry experiments, staining was performed as previously described [60]. Cells were permeabilized in 0.1% Triton X-100 for 20 min, washed, and blocked with 10% donkey serum for 1 h at room temperature. Primary antibodies were diluted in 10% donkey serum, which included rabbit anti-α-SMA (1:200, Abcam, Waltham, MA, USA), mouse anti-MPZ (1:100, conjugated to Alexa Fluor 647, BioLegend, San Diego, CA, USA), rabbit anti-P75 (1:400, Promega, Madison, WI, USA), mouse anti-tubulin β3 (Tuj1, 1:400, conjugated to Alexa Fluor 488, BioLegend), and mouse anti-tubulin β3 (Tuj1, 1:400, conjugated to Alexa Fluor 647, BioLegend). Secondary antibodies were diluted in 10% donkey serum, which included donkey anti-rabbit IgG (1:500, conjugated to Alexa Fluor 546, Invitrogen) and donkey anti-rabbit IgG (1:500, conjugated to Alexa Fluor 647, Invitrogen). Cell nuclei were counterstained with DAPI solution (Invitrogen) and mounted on slides with Aquapoly/mount (Fisher Scientific Polysciences, Inc., Pittsburgh, PA, USA).

### 4.8. Immunohistochemistry for In Vivo Experiments

Following in vivo neurosphere transplants, mice were sacrificed by CO_2_ asphyxiation. The colon was dissected and opened along the mesenteric border, and the mucosa and submucosa were removed to expose the longitudinal muscle myenteric plexus (LMMP), as previously described [61]. LMMP samples were fixed in 4% paraformaldehyde overnight at 4 °C.

Wholemount staining of LMMP tissues was performed as previously reported [62,63]. Tissues were incubated with blocking buffer containing 10% donkey serum, 10% bovine serum albumin, and 1% Triton X-100 for 1 h at room temperature. Primary antibodies were diluted in this blocking buffer, which included mouse anti-MPZ (1:100, conjugated to Alexa Fluor 647, BioLegend), rabbit anti-P75 (1:400, Promega), and mouse anti-tubulin β3 (Tuj1, 1:400, conjugated to Alexa Fluor 488, BioLegend). Secondary antibodies were diluted in blocking buffer, which included anti-rabbit IgG (1:500, conjugated to Alexa Fluor 647, Invitrogen). Cell nuclei were counterstained with DAPI solution (Invitrogen) and mounted on slides with Aquapoly/mount (Fisher Scientific Polysciences, Inc.).

### 4.9. Imaging

Images were obtained with a Keyence BZX-700 All-In-One microscope (Keyence America, Itasca, IL, USA), a Leica MZ FLIII fluorescence stereomicroscope (Leica Microsystems, Deerfield, IL, USA), a Nikon Eclipse 80i upright fluorescent microscope (Nikon Instruments, Melville, NY, USA), or a Zeiss LSM 800 Airyscan confocal microscope (Carl Zeiss Meditec, Dublin, CA, USA). Several images of Alexa Fluor 647 fluorescence were pseudocolored green for greater visual distinction with tdT. Images of neurospheres were analyzed using ImageJ software v1.53t (National Institutes of Health, Bethesda, MD, USA) and their size quantified through binary thresholding (Huang method) to designate an area of interest. 3D-rendered images of confocal Z-stacks were produced using the *Volume Viewer 2.01* plugin of ImageJ.

### 4.10. Statistical Analysis

Data were analyzed using GraphPad Prism v9 (GraphPad Software, Inc., San Diego, CA, USA). One-way analysis of variance (ANOVA) with Welch’s corrections was performed on data with multiple groups. Gene expression data were analyzed using a one-sample *t*-test of the Log^2^ fold change values (LogFC) between neurospheres normalized to gene expression in fibers, with a hypothetical mean value of 0 LogFC (unchanged). For all comparisons, *p* < 0.05 indicated statistical significance. Data are shown as means ± SEM unless explicitly stated otherwise.

## Data Availability

All data are available upon request to the corresponding author.
